# Accelerometer-Based Physical Activity Levels Differ between Week and Weekend Days in British Preschool Children

**DOI:** 10.3390/jfmk4030065

**Published:** 2019-09-12

**Authors:** Clare M. P. Roscoe, Rob S. James, Michael J. Duncan

**Affiliations:** 1 Human Sciences Research Centre, University of Derby, Kedleston Road, Derby DE22 1GB, UK; 2 Centre for Applied Biological and Exercise Sciences, Coventry University, Priory Street, Coventry CV1 5FB, UK; aa8396@coventry.ac.uk (R.S.J.); apx214@coventry.ac.uk (M.J.D.)

**Keywords:** physical activity, preschool children, health promotion

## Abstract

Participation in physical activity (PA) is fundamental to children’s future health. Studies examining the temporal pattern of PA between weekdays and weekends in British preschool children are lacking. Therefore, the aim of this study was to compare PA levels between week and weekend days for UK preschool children, using objective measurements. One hundred and eighty-five preschool children (99 boys, 86 girls, aged 4–5 years), from central England wore a triaxial accelerometer (GENEActiv) for 4 days to determine PA. The time (min) and percentage (%) of time spent in light, moderate and vigorous PA (MVPA) was determined using specific cut-points for counts per minute related to 3–5 year olds. Of the sample, none of the children met the UK recommended 180 min or more of PA per day. A significant difference (*P* < 0.05) was observed between the amount of time that preschool children spent in sedentary behaviours on weekdays (91.9%) compared to weekend days (96.9%). During weekdays and weekend days, 6.3% and 2.0% of time was spent in MVPA, respectively. Therefore, a substantial proportion of British preschool children’s day is spent in sedentary behaviours, with less MVPA accrued during the weekend. Regular engagement during the weekdays provides opportunities to accrue PA, which may not be present on weekend days.

## 1. Introduction

Physical activity (PA) during preschool years is critical to a child’s development and overall health and well-being [1,2]; therefore, it is important to integrate PA into early childhood [3,4]. In 2016, over 41 million children worldwide under the age of 5 years were estimated to be overweight [5]. Childhood obesity is an increasing public health concern [6] and weight gained by the age of 5 years has been reported as a predictor of being overweight in adulthood [7]. Physical activity levels and sedentary behaviours of children in the UK have been viewed as ‘obesogenic’ [8,9], with habitual PA declining over recent years and sedentary behaviour being the dominant state of children’s PA levels during their preschool day [4,10,11,12]. Although studies have examined PA in children aged 5 years and above, fewer studies have been conducted with preschool children. This limited evidence base in UK preschool children’s PA levels is therefore a cause for concern. 

It has been recommended that preschool children in the UK should ideally be participating in at least 180 min of PA per day [13,14,15]. Studies have discovered that preschool children spend the majority of their day in sedentary behaviours and a low proportion of their day in moderate to vigorous PA (MVPA) (<15%) [10,16,17]. Of children aged between 2 and 4 years in England, only about one in 10 meet the recommendations of at least 180 min of PA per day [18,19]. It has been reported that children engaged in 7.7 min of MVPA per hour at preschool [20]. Therefore, in accordance with Pate et al. [20], if a child, for example, attends preschool for 8 h, they would only engage in ~1 h of MVPA and it is unlikely they would participate in a further 2 h of PA outside of preschool. However, no study has systematically checked to see whether there is a difference in physical activity between weekdays, when the child attends preschool, and weekend days, when the child is influenced more by their home environment. O’Dwyer et al. [21] reported that there were discrete periods during the after-preschool hours and at the weekend when PA levels were low, yet children who attended preschool for full days engaged in 11.1 min MVPA less than those attending for half days, suggesting that the preschool environment is related to decreased PA. That said, studies have shown that PA in preschoolers differs over the course of the day and the week in countries such as Sweden, England and Denmark [21,22,23,24], with some studies reporting that preschool children are more often physically active on weekend days than on weekdays in Australia and England, for example [21,25], while others found that preschool children undertook more total PA and MVPA during preschool hours in Sweden, Denmark, England and Finland [22,24,26,27]. Therefore, additional research is required to identify any potential differences in PA between weekdays and weekend days in preschool children. 

The accurate measurement of PA is fundamental in evaluating the effectiveness of interventions and understanding relationships between PA and health [28]. Measuring habitual PA accurately is beneficial when observing the frequency and distribution of PA in preschool children and identifying the amount of PA that could influence their health. The objective monitoring of PA is important and accelerometers have become a reliable and valid way of estimating children’s PA [29,30], whilst also showing promise in monitoring preschool children’s PA. Accelerometers are an appropriate objective measure in terms of validity, reliability and practicality as a method for the measurement of intensity, duration and frequency of movement for sedentary behaviour and habitual PA in 3–5 year olds [1,31,32,33]. Accelerometers can be set at different sampling intervals, with some studies being set at one-minute intervals [1,34]. However, one-minute sampling intervals may mask the short intermittent bursts of activity that are representative of young children and therefore shorter sampling intervals have been recommended [20,35]. As very few studies have used objective monitoring of PA via accelerometery in preschool children, then further research is required to examine the intensity of PA that these children participate in on weekdays in preschool and at the weekend.

This is the first study to compare PA levels between week and weekend days, using objective measurements in the form of the newly calibrated GENEActiv accelerometer cut-points for preschool children in the UK. This study aims to determine whether the intensity and duration of PA varies between weekdays and weekend days. 

## 2. Materials and Methods

### 2.1. Participants and Data Collection

Participants in this study were preschool aged children from 11 preschools in North Warwickshire, England. This study was completed in the Nuneaton and Bedworth Borough, which is in the top 10% of the most deprived Super Output Area’s in England on the Index of Multiple Deprivation (IMD) and is ranked as the 111th most deprived Local Authority District out of 326 in England [36]. Ethics approval (P45654) was granted by the Faculty of Health and Life Sciences Ethics Committee, Coventry University and parental consent was obtained. The participants were a convenience sample and included 185 preschool children (99 boys, 86 girls), aged 3–4 years, from a deprived area.

### 2.2. Anthropometric Assessment

Height was measured to the nearest mm, in bare or sock feet, using a standard portable stadiometer (Leicester height measure, Leicester, UK). Body mass was measured to the nearest 0.1 kg using portable weighing scales (Tanita scales, Tokyo, Japan); the children were lightly dressed (t-shirt and light trousers/skirt) and barefoot or in socks. The measurements were repeated twice and the average score was recorded. Body mass index (BMI) was calculated as kg/m^2^ and weight status was categorised as overweight/obese or normal weight using standardised international cut-points [37]. 

### 2.3. Assessment of Physical Activity

Daily total PA was measured using a GENEActiv waveform triaxial accelerometer (ActivInsights Ltd., Kimbolton, UK). The accelerometer measured at 10 epochs (s) and a sample frequency of 100 Hz, so as to enable an accurate assessment of the intermittent activities of preschool children [38,39,40]. The GENEA accelerometer was attached using a watch strap and positioned over the dorsal aspect of the right wrist, midway between the radial and ulnar styloid process. Accelerometers worn on the wrist are more convenient to wear and lead to greater compliance during prolonged wearing when assessing habitual activity [41]. The participants wore the accelerometers for four consecutive days; this included two weekdays in the setting and two weekend days. Each child was required to wear the accelerometer for a minimum of 6 h per day to be included in the study, although it was preferred that they wore them at all times. All children received a letter to take home describing how and when they should wear the GENEActiv accelerometers. Non-wear time was defined as 90-minute windows of consecutive zero or nonzero counts [42]. “Nonzero” counts are caused by artefactual accelerometer movements during non-wear periods—for example, accidental movement of the accelerometer, such as the device being nudged when on a bedside table [42]. The 90-minute window was chosen as this was found to better predict time spent in sedentary behaviours and PA levels [42]. This said, Esliger et al. [43] suggested that a period of 20 min of consecutive zero counts is appropriate for children, as motionless bouts of ≥20 min are biologically implausible. However, it was reported that this low threshold causes an unrealistically high number of non-wear periods [44]. Therefore, it was recommended to use 90 minute consecutive zero counts, as this prevents the overestimation of non-wear time and the underestimation of sedentary behaviours in overweight to obese children [42]. The amount of wear time and percentage (%) of wear time that each child spent in different intensities of PA was calculated for weekdays and weekend days. It is recommended that four days, including one weekend day, is appropriate for measuring habitual PA [45]. Given the logistics of ensuring that children aged 3 years of age wore the accelerometer for the whole monitoring period, participants were included in the final data analysis providing they had worn the accelerometer for 3 days (when in the setting and at the weekend) and for a minimum of six hours each day, similar to previous research [46,47,48]. Of the 185 sample, 178 children’s accelerometer data were recorded; data for seven children were not useable. This was due to the children either not wearing the GENEActiv accelerometers or technical difficulties with the accelerometers or recording of the data. The final sample included in the analysis was 178 children (95 boys, 83 girls), aged 3–4 years. Only four out of the 178 participants were full-time; the remainder were part-time nursery attendees. 

For each of the epochs (number of seconds), movement data (activity counts) were added and logged; these were then processed and analysed. Accumulated activity counts were categorised in terms of intensity such as sedentary behaviour, light, moderate and vigorous PA [1]. Cut-points for sedentary behaviour, light PA and moderate and vigorous PA were used to determine the PA intensity of the preschool children. The cut-points used were determined specifically for children aged 4–5 years using GENEA accelerometers, albeit they were calibrated in a laboratory-based study; they are the most relevant cut-points for the preschool children’s age in this study (3–4 years) as they are the closest cut-points that are calibrated and reported in the literature [49]. The difference in age should have very little impact on the results, as they are as closely aligned in age as possible and 4 year olds/preschool children have been used for the calibration and ultimately are assessed in this study. The preschool children in the laboratory-based study that was used to determine cut-points for this current study completed six activities, which ranged from lying supine to running. They wore the GENEA accelerometers on both their left and right wrists and used a Cortex mask for gas analysis; VO_2_ data were used to assess criterion validity [49]. The cut-points determined were as follows: dominant hand <8.1 cpm for sedentary activity, 8.1–9.3 cpm for light activity and 9.3+ cpm for moderate and vigorous PA. For the non-dominant hand, the cut-points were <5.3 cpm for sedentary activity, 5.3–8.6 cpm for light activity and 8.6+ cpm for moderate and vigorous PA [49]. On the accelerometers, the ‘Epoch Converter’ creates epochs of 1, 5, 10, 15, 30 or 60 s; the means that for each parameter, the Sum Vector Magnitude is calculated for each epoch [50]. Children were classified as either meeting (sufficiently active) or not meeting (insufficiently active) the requirement of 180 min per day of PA for 0–5 year olds. 

### 2.4. Statistical Analysis

The percentage of time in sedentary behaviour, light PA and MVPA was determined, as was the mean amount of time (min) spent in sedentary behaviour, light PA and MVPA, during week and weekend days. Each data set was tested for skewness and kurtosis. Arcsine or inverse data transformation techniques were then used on any data set that did not have a normal distribution, as follows: mean time sedentary for the week and weekend (arcsine transformation); MVPA at the weekend (inverse transformation); percentage time for sedentary behaviour at the weekend (inverse transformation); and MVPA at the weekend (inverse transformation). Any differences in PA due to sex or day of the week were analysed using a series (separate ANCOVA for each category of PA) of 2 (weekday vs. weekend) × 2 (sex) repeated measures analysis of covariance (ANCOVA) controlling for wear time. The Statistical Package for Social Sciences (Version 22, SPSS Inc., Chicago, Ill, USA) was used for statistical analysis and the alpha level was set a priori at *P* = 0.05.

## 3. Results

Descriptive characteristics, including mean time (min) spent in the different intensities of PA during the week and weekend days, are summarised in Table 1. Of the sample, none of the 178 children met the UK recommended 180 min or more of PA (light, moderate and vigorous intensity) per day. Two, circa 1%, of the children did meet the 180 mins on one of their days, but not on all days.

**Figure 1:**
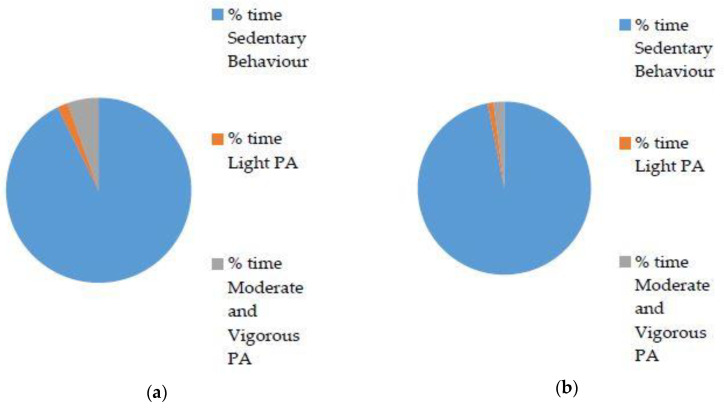
Percentage (%) daily time spent in different intensities of physical activity. (**a**) Weekdays and (**b**) weekends for 178 preschool children.

Preschool children respectively spent 91.9% and 1.8% of time in sedentary behaviour and light PA on weekdays, and 96.9% and 1.1% of time in sedentary behaviour and light PA at the weekend. During weekdays and weekend days, 6.3% and 2.0% of time was spent in moderate and vigorous PA, respectively. The percentage (%) of daily time spent in different intensities of PA during weekday and weekends for preschool children can be viewed in Figure 1. 

There was a significant difference in the percentage of time (relative) spent in sedentary behaviour between week and weekend days (*P* < 0.05), yet no differences were found between week and weekend days for light or MVPA (Figure 1). There was a significantly smaller mean time in minutes spent in sedentary PA (mean difference = 91.874, *P* = 0.001) during weekdays compared to weekends. This pattern was reversed for moderate and vigorous PA (mean difference = 4.545, *P* = 0.001), with a larger mean time spent in vigorous PA during weekdays compared to weekend days. Wear time had no effect on PA, as there was no significant interaction between wear time and weekday (F = 1.308, *P* = 0.257) or between wear time and weekend day (F = 1.107, *P* = 0.297) for the significant difference reported for the percentage of time (relative) in sedentary behaviour. Sex had no significant effect on any PA intensity (all *P* > 0.05).

## 4. Discussion

The current study sought to compare PA levels of preschool children between weekdays and weekend days, and the key finding of this study is that there are significant differences in PA between weekdays and weekend days. Of particular note, more than 90% of the time during both weekdays and weekend days was spent in sedentary behaviour. Additionally, this study found that none of the children were considered ‘sufficiently active’, failing to participate in the UK recommended level of at least 180 min of light PA and MVPA per day of total PA for health. As zero preschool children in this study reported meeting the PA guidelines, this is a major concern, especially as the children were struggling to achieve 60 min of total PA. As the majority of this sample (98%) were part-time preschool children, this could be reflective of this specific preschool child. The children in this study spent time in both the preschool setting and with their parents, in the home environment. It would be pertinent to assess preschool children who are full-time, to ascertain if this provides a similar or different outcome in their PA levels. This would be influential in identifying whether the results of this study are reflective of British preschool children, or if the time children spent in preschool affects their PA levels. 

Studies have shown that parents have a significant impact on PA in their preschool children on week and weekend days [51,52,53,54]. Therefore, other explanations for the differences in sedentary behaviour between the week and weekend days could be a result of parents displaying higher sedentary behaviours when they are with their children, and children who are exposed to these behaviours copy them during weekend days [55]. This is supported by Sigmundová et al. [56], who found that children from both urban and rural populations had a stronger significant association with sedentary behaviour during weekend days as compared to weekdays. Moore et al. [57] discovered that middle-class American children aged 4–7 years old (incorporating the preschool years), who have one physically active parent, had a relative odds ratio of their child being active between 2.0 (mother) and 3.5 (father). However, if both parents were active then the relative odds ratio was 5.8, with no difference reported between week versus weekend day. Sigmundová et al. [56] monitored children from both urban and rural areas, and discovered that if mothers are more active, then their children are more likely to be more physically active. This was observed to be significant only on weekend days. We do not know the activity patterns of the parents of the children who took part in the present study, but this may have been a contributing factor to the sedentary behaviour patterns that were reported.

A second key finding of this present study is that there are differences in the percentage of time spent in different intensities of PA between weekdays and weekend days for preschool children. During weekdays, the children spent significantly less time in sedentary behaviours (91.9% vs. 96.9%), when compared to weekends. This finding was recorded using the right wrist, which in this study was the dominant hand for 169 of the children (91.4% of the sample). This finding contradicts research by Vásquez et al. [58] in Chilean children (North of Santiago City), who objectively measured PA via a Tritrac-R3D research ergometer and reported that preschool children spent more time in sedentary activities in day-care centres (week vs. weekend) and the children were more active at home at weekends. These differences were also linked with the children’s diet and it was discovered that the energy balance was appropriate during the week, as the energy intake in the preschools was reduced. This could explain the differences, as the day-care settings were providing less energy intake; therefore, the children may have been less inclined to be active. Equally, these findings could have been representative of the cultural background, geographical location or differences in the method used for objective measurement of the children involved. A further reason for potential differences between the sedentary behaviours of preschool children during the week and weekend days could be attributed to the time that children spend watching television or playing on computer games, smart phones/tablets (screen time). Previous research has shown that Australian preschool children from different socio-economic backgrounds whose parents limit television viewing spent significantly less time in sedentary behaviours [59]. A study of preschool children from a mixed socio-economic area in the southwest of England reported that 12% of boys and 8% of girls watched ≥2 hours of TV each weekday, compared to 45% of boys and 43% of girls who watched ≥2 h a day on weekend days [60]. The amount of screen time that children in this current study participated in could have been a contributing factor to the differences in time spent in sedentary behaviours during week and weekend days. Parental influence on PA during weekend days may therefore be an important factor that requires greater attention by public health professionals; this could be a result of parents lacking an understanding of appropriate PA to deliver to their children, or a lack of time. Despite this, the extant literature on parental influence on PA levels in British preschool children is scarce. Additional work is required on this topic, in the context of weekday to weekend day variations in children’s PA. Equally, the intensity levels of PA of preschool children could be improved through interventions, both in preschools and at home with parents.

Research has reported PA levels and sedentary time as being highly varied and inconsistent between studies across different countries, making it hard to determine preschool children’s true PA levels and sedentary behaviour [61]. Reilly [12] measured PA levels from studies over the period of 2000–2008 and discovered that sedentary behaviour was particularly high. However, more recently, in a data collection period from 2006–2009 and a 7 month data collection period in the year 2013, it was reported that 100% of UK preschool children met the recommend daily PA guidelines [22,23]. The current data from this study show that a substantial proportion of each day is spent in sedentary behaviour in British preschool children from a deprived area. This finding has important public health implications around the excessively high sedentary behaviours displayed by preschool children in the UK and therefore provides a clear indication that interventions aiming to convert sedentary behaviours into light or MVPA are required. These data were obtained across a wide measurement period and across all seasons and therefore provide a spread of representative data for the whole of the year. However, this study did not assess each child at different points throughout the year, as it is very labour-intensive and demanding to assess PA in preschool children at one time point and then assess them again across different seasons, and would likely lead to much higher attrition. Therefore, this study did not consider seasonal adjustments. However, future research may consider this, to identify if preschool children are more active in the summer months in England, when compared to the cold environment in the winter months, similar to the results found in 437 preschool children in America [62]. However, this was in contrast to a study of 214 children aged 3–5 years in America, which reported no differences in PA levels between the summer and autumn months [63]. Moreover, further studies in Sweden and America [64,65] also found no variation according to the season of measurement in preschool children. As none of these studies were conducted in England, future research into the seasonal variation of PA in England would be beneficial. 

The amount of time spent in different intensities of PA was found to vary between weekdays and weekends, with less moderate and vigorous PA accrued during weekends. However, MVPA was very limited in both parts of the week. Such a finding might be suggestive that regular engagement in the preschool environment provides greater opportunities to accrue PA, which may not be present in the home setting. The light intensity minutes were very low during both the week and weekend days. The wrist-worn accelerometers may not be very precise at detailing light ambulation, where playing with Lego® was at the top end of the sedentary category. Therefore, future research to determine the accuracy of the light PA classifications would be beneficial. Similar research using Actigraphs has shown that using the cut-point of 160 cpm to distinguish light intensity from sedentary behaviours is questionable [66]. It is believed that this threshold may misclassify sedentary behaviours such as seated play and crafts as light intensity, which would cause an overestimation of total PA minutes and underestimate the steps per day [66] that are required to achieve the daily UK 180 minutes of recommended PA. This is a current problem, as there is no consensus on the optimal cut-points for distinguishing sedentary from light PA in preschool children [56]. Also, the cut-points used for light intensity PA in this study were taken from laboratory-derived tasks that were constant in nature, whereas the real-life activities of preschool children are more varied and intermittent. This may have made it more difficult to differentiate light PA from sedentary behaviour, as the determination is dependent on the cut-point used. However, this is a feature of most of the research using accelerometers to classify/assess PA [66,67]. Therefore, it could be suggested that the cut-points used in this study were too conservative, however, as they are the only cut-points related to preschool children specifically for the GENEActiv accelerometers, then they were the most appropriate to have adopted. 

Using wrist-worn accelerometers can be logistically and practically challenging with preschool children [31], as the accelerometer can sometimes be regarded as uncomfortable or an annoyance when worn for long periods of time, thus questioning the appropriateness of the accelerometer for preschool children. In the current study, although a cut-off of 6 h per day was employed for inclusion in the data analysis, the participants exceeded this value with total mean wear time per day for all days, which was over 577 min (>9.6 h). The mean wear time for weekdays was 573 min (9.6 h) and that for weekend days was 581 min (>9.7 h). We took this to be an indication that the majority of the children in this study were comfortable wearing the accelerometers. It could be questioned whether <600 min/day (10 h) is a true representation of a preschool child’s whole day (24 h). It would be beneficial for future research to measure PA for a greater duration, for example 12 hours, to see if this affects the total PA in a day, in terms of less sedentary behaviours and more MVPA. As mentioned, it could be questioned whether wearing the accelerometer on the wrist is suitable for preschool children. Research has looked at the difference between wearing a wrist and hip accelerometer on preschool children and one study in Scotland found that wrist-worn accelerometers provided a valid estimate of total physical activity, whereas hip-worn accelerometers showed a reasonable agreement to cut-points [68]. This was supported by a study in Stockholm, Sweden, which similarly monitored preschool children and found that wrist-worn accelerometers performed more accurately when assessing time spent in sedentary, light activity and MVPA, when compared to hip-worn accelerometers [69]. In terms a study by Johannsson et al. [69], however, there were stark differences between the mean (SD) counts measured over 5 s for wrist and hip activity, with the vector magnitude (a combined measure of the three axes, x, y and z) when watching a cartoon measuring 91 (73) for the wrist and 14 (15) for the hip, and when dancing, the 1093 (330) was measured for the wrist and 396 (148) for the hip. These are large contrasts, highlighting that where the accelerometers are worn is an important factor to be considered. This present study had the preschool children wearing them on their wrist, which, in accordance with other studies [68,69], is the more accurate and valid place to wear them when using the vector magnitude measure, as opposed to the vertical axis measure.

Although the current study successfully used accelerometery as an objective monitoring tool in preschool children, some limitations should also be considered. Some of the accelerometers failed to record data; this manufacturer problem caused no data to be recorded for seven participants. GENEActiv accelerometers worn at the wrist may not be capable of detailing light ambulation precisely, as possibly indicated by the low levels of light PA reported. Equally, wrist-worn accelerometers in young children may also impact the light intensity PA data; however, the research does appear to show that the location, whether at the wrist or hip, has no significant effect on the PA levels reported [70,71]. As previously stated, the preschool children were assessed once in a season and not across all seasons. Although children were assessed throughout different seasons, which provided representative data, the lack of assessment of each child in all seasons should be considered a limitation of the study. The preschool children that were monitored were drawn from a deprived part of the UK. It has been reported that the prevalence of obesity amongst 4–5 year olds in the most deprived 10% of England is approximately double the levels of the least deprived 10% of England [72]. In this current study, 10.8% (20 out of 185) were considered obese. Low socio-economic status (SES) children face greater barriers to becoming physically active and, as they age, low SES individuals have higher rates of obesity and associated comorbidities [73]. Additionally, people from lower SES groups predominantly live in areas that do not support walking and cycling [74]. This viewpoint suggests that deprived areas do not facilitate PA as effectively as other areas and, as such, people living in deprived areas may not participate in PA as frequently. This said, further research comparing both high and low socio-economic status groups would be welcome to extend the literature on preschool children. Understanding the levels of PA in this group is useful to allow for the planning of early interventions to improve current and future health. 

## 5. Conclusions

The current study is the first to objectively compare PA levels between weekdays and weekend days in preschool children in the UK using GENEActiv accelerometers, and one of the first to report objectively monitored PA levels of preschool children from deprived areas in the UK. The results of this study suggest that none of the preschool children in this sample achieved the UK recommended guidelines of PA for health. Additionally, and of great concern, it was found that preschool children spend 90% of their time engaged in sedentary behaviours. This study indicates that preschool children participate in more MVPA during weekdays compared to weekend days; however, participation in MVPA was minimal throughout the week. This information can help to promote future interventions that focus on enhancing PA and encouraging participation in LPA and MVPA during both week and weekend days, so as to improve physical development and a healthy weight status in preschool children.

## Figures and Tables

**Table 1:** Children’s descriptive characteristics. Data represent mean ± SD, *n* = 178.

Characteristics		Values
Age (years)		3.4 ± 0.5
Mass (kg)		16.8 ± 2.5
Height (cm)		101.7 ± 4.8
Body mass index (kg/m^2^)		16.3 ± 1.9
Waist circumference (cm)		55.0 ± 3.9
Mean wear time (min) during the week		572.0 ± 99.0
Mean wear time (min) during the weekend		581.0 ± 126.0
Mean sedentary behaviour (min) during the week		527.0 ± 94.0
Mean sedentary behaviour (min) during the weekend		565.0 ± 117.0
Mean light physical activity (PA) (min) during the week		10.0 ± 15.0
Mean light PA (min) during the weekend		7.0 ± 10.0
Mean moderate and vigorous PA (MVPA) (min) during the week		36.0 ± 22.0
Mean moderate and vigorous PA (min) during the weekend		12.0 ± 9.0
Sedentary behaviour (%) during the week		91.9 ± 4.3
Sedentary behaviour (%) during the weekend		96.9 ± 2.0
Light PA (%) during the week		1.8 ± 2.4
Light PA (%) during the weekend		1.1 ± 1.5
MVPA (%) during the week		6.3 ± 3.6
MVPA (%) during the weekend		2.0 ± 1.6
Met PA guidelines of at least 180 min per day total PA (%)	Sufficiently Active Insufficiently Active	0 100
